# Blisters on graphite surface: a scanning microwave microscopy investigation

**DOI:** 10.1039/c9ra04667d

**Published:** 2019-07-26

**Authors:** Eleonora Pavoni, Rossella Yivlialin, Christopher Hardly Joseph, Gianluca Fabi, Davide Mencarelli, Luca Pierantoni, Gianlorenzo Bussetti, Marco Farina

**Affiliations:** Department of Information Engineering, Università Politecnica delle Marche Ancona Italy e.pavoni@staff.univpm.it; Department of Physics, Politecnico di Milano p.za Leonardo da Vinci 32 I-20133 Milano Italy

## Abstract

Scanning microwave microscopy (SMM) is based on the interaction between a sample and an electromagnetic evanescent field, in the microwave frequency range. SMM is usually coupled with a scanning probe microscopy (SPM) technique such as in our case, a scanning tunneling microscope (STM). In this way, the STM tip is used to control the distance between the probe and the sample while acting as an antenna for the microwave field. Thanks to the peculiarity of our home-made setup, the SMM is a suitable method to study blisters formed on HOPG surface as consequence of an electrochemical treatment. Our system has a “broad-band” approach that opens the way to perform local microwave spectroscopy over a broad frequency range. Moreover, microwaves have the ability to penetrate into the sample allowing the sub-surface characterization of materials. The application of the SMM to characterize blisters formed on the HOPG surface provides information on the sub-layer structures.

## Abstract 2

Blisters formed on an electrochemically treated HOPG are investigated by Scanning Microwave Microscopy that allows a spectroscopic and sub-surface characterization.

## Graphical abstract

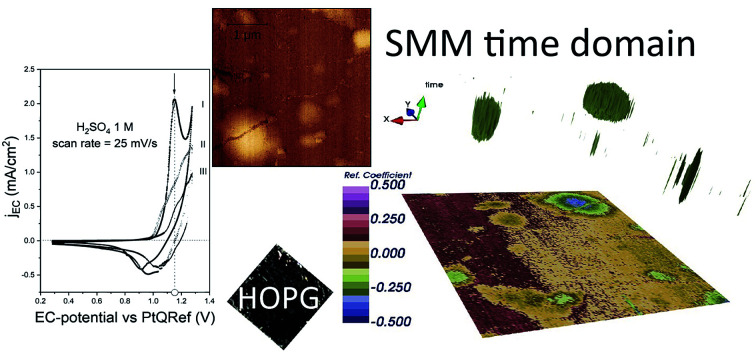

## Introduction

The scanning microwave microscope (SMM) evaluates the interaction between a sample and an electromagnetic evanescent field, in the microwave frequency range. In order to control accurately the probe–sample distance, SMM is coupled with a scanning probe microscopy (SPM) technique, such as atomic force microscope (AFM) or, in our case, scanning tunnelling microscopy (STM).^1,2^

The STM assisted SMM makes use of a high frequency microwave signal, which is injected from the Vector Network Analyser (VNA) through a coaxial connector terminated with a sharp metallic tip. In this way, the metallic tip has a double function: it works as a small monopole antenna while collecting the tunnelling current. The tip–sample distance should be kept smaller (or at least comparable) to the characteristic size of the tip apex. As a matter of fact, a nanometric probe–sample distance is required in order to achieve an evanescent field (the electromagnetic field decays exponentially from the probe), a good sensitivity and an atomic scale spatial resolution (since near a thin edge the electromagnetic field is almost singular).^3,4^

Generally, the SMM measurements are performed at a single frequency by using a resonator as an impedance matching system, so that, to gain high sensitivity, the user has to work only at the resonance frequency.^5–8^

With our SMM implementation, so-called “broad-band” approach, no resonator is needed; in this way, the sample is studied by using a large range of frequencies and it is possible to perform local microwave spectroscopy.^9^ Two of the important aspects that contribute to the high sensitivity of our instrumentation are: (i) the correlation of the images obtained at several close frequencies (broad band analysis) and (ii) the introduction of time-domain reflectometry. This latter makes use of an inverse Fourier-transform applied to the frequency domain microwave data. In fact, during the SMM scan a 3D matrix is recorded: a typical image is 256 × 256 spatial points (*x* and *y* coordinates) over 512 microwave frequency points (working band). The acquired data are the reflection coefficients over a given frequency range, *i.e.* the ratio between the reflected and the incident signals; the signal is injected and collected by the VNA and reflected by the sample. By the inverse Fourier-transform, we obtain a description of how the reflected signal changes with time, mimicking a kind of measurement known as ‘Time-Domain Reflectometry’, used in ground penetrating radar or in signal integrity applications.^10^

It is well known that microwaves have the ability to penetrate to some extent into the samples, allowing the sub-surface characterization of materials; the penetration depth could reach a hundred nanometers depending, mainly, on the nature of the material and on the frequency used.^11,12^

This property can be usefully exploited to gain more information on the sub-surface environment existing in electrochemically intercalated systems, like graphite compounds. Among these, typically highly oriented pyrolytic graphite (HOPG) is used as a working-electrode for electrochemistry-based applications (*e.g.* batteries and hydrogen storage materials) and graphene production.^13–16^ When HOPG is immersed in an acid electrolyte solution, a solvated anion intercalation occurs at well-known applied potentials.^17^ The presence of water molecules inside the stratified graphite structure induces the evolution of gases (namely CO, CO_2_ and O_2_) and a consequent swelling of the crystal surface (blister).^18,19^ Blister formation and growth has been the topic of many researches in the last twenty years.^14,17–23^ Nonetheless, few information have been collected regarding the underneath graphite layers after the anion intercalation. An important result was obtained at the end of nineties by Alliata and co-workers^22^ by comparing the different height between neighbour graphite terraces; the authors succeeded in giving an estimation of the depth of anion intercalation inside the crystal. However, a spectroscopic analysis of the blistered sample below the uppermost layer is still missed. This kind of investigation could provide some advisable knowledge for the production of massive graphene by the electrochemical intercalation,^14,18,24^ where the status of the entire graphite crystal strongly determines the quality of the obtained graphene flakes.

This paper aims to characterize intercalated HOPG, *i.e.* where blisters are formed on the surface of graphite, as a consequence of the electrochemical process, by using the scanning microwave microscope. The improvement that can be obtained with such microscopy regards not only the imaging, but also the needless of graphical post-processing. Moreover, the SMM allows a spectroscopic and a sub-surface characterization of the graphite that are helpful for a better understating of the above described phenomena.

## Experimental

A z-grade HOPG sample (Optigraph) was immersed inside a three-electrode electrochemical cell to induce the anion intercalation. The cell exploits two Pt wires: the biggest one represents the counter electrode, where the reduction (oxidation) process occurs when the sample (the working electrode) undergoes an oxidation (reduction) reaction. A second Pt wire is used as a reference and only the apex of the wire is immersed inside the cell. A Pt wire is not a real reference electrode but, within small potential range and with some acid electrolytes, it shows both a good stability (few tens of millivolts) and a fix shift (+743 mV) with respect to the standard hydrogen reference electrode. A 1 M H_2_SO_4_ electrolyte was purified by bubbling pure Ar in the solution for some hours. The cyclic-voltammetry (CV) was performed several times between 0.3–1.3 V, where the main intercalation features are placed. The sample surface morphology was checked by atomic force microscopy (AFM) in tapping mode. The electrochemical sample preparation and its morphological analysis were obtained by a commercial Keysight 5500 EC-AFM system.

**Fig. 1:**
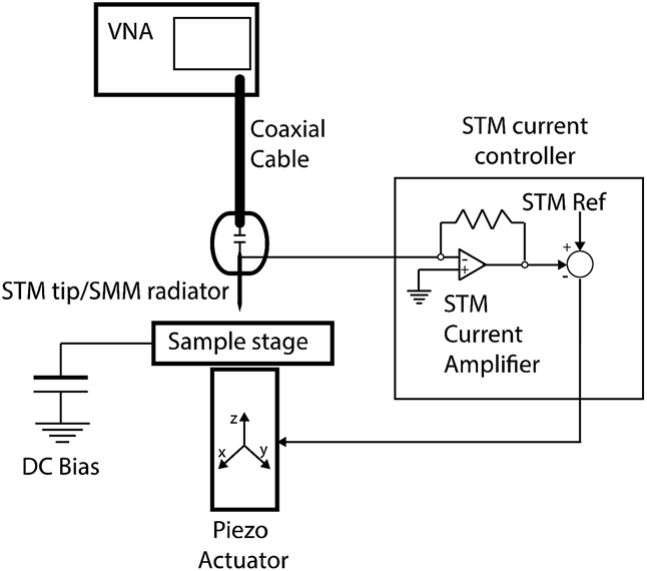
Schematic representation of our home-made STM-assisted SMM system.

Fig. 1 illustrates schematically our homemade STM-assisted SMM; the system is composed of an NT-MDT Solver Pro P-47 scanning probe microscope with a custom-designed probe head featuring a high-sensitivity current amplifier for STM operation. A metallic tip (made of Pt/Ir) is used as SMM probe/antenna and it simultaneously monitors the tunnelling current (STM); in this way the tunnelling circuitry is used to keep the suitable (few nanometres) tip–sample distance. The STM probe is capacitively coupled to a Keysight PNA E8361A 67-GHz VNA *via* a flexible coaxial cable.

The scans were obtained by using a bias of 0.1 V between the HOPG and the probe and by setting the value of the tunneling current at 0.05 nA. STM images were corrected to remove the sample tilt, while no such removal was necessary for SMM images.

## Results and discussion

**Fig. 2:**
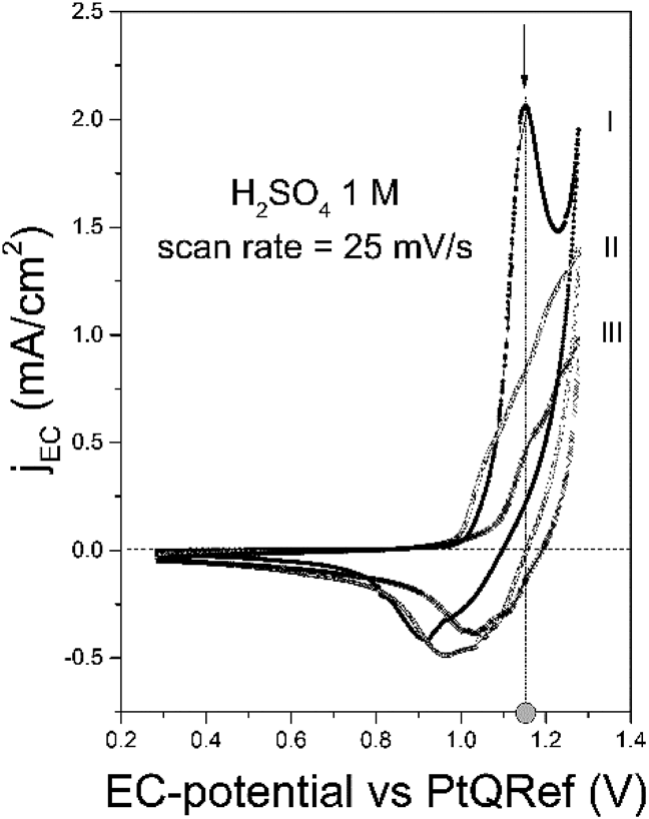
Three subsequent cyclic-voltammetries acquired on the graphite immersed in sulphuric electrolyte. Both the intercalation potential (grey-filled circle) and the current feature (arrow) are marked in the image.

In Fig. 2 (line labeled I), we report the collected CV of graphite in 1 M H_2_SO_4_. At around 1.0 V the faradaic current shows an enhancement, which corresponds to the oxygen evolution reaction (OER).^25^ The intercalation potential is labeled in the figure (gray-filled circle) and, at this value, a feature in the voltammogram (arrow) is observed. This ensures that the anion intercalation occurs and blisters affect the sample surface.^18,19,26^

To ensure a significant damage of the pristine graphite surface, the CV can be repeated a few times (two in this example, lines labelled as II and III). Despite a similar line shape and the presence of shoulders at the intercalation potential, the faradaic current reduces after each cycle. This effect is caused by a not complete de-intercalation process during the cathodic regime (detectable by the presence of a negative peak): during a following cycle, part of the graphite inter-layer space is still filled with anions which preclude the intercalation of other compounds.

**Fig. 3:**
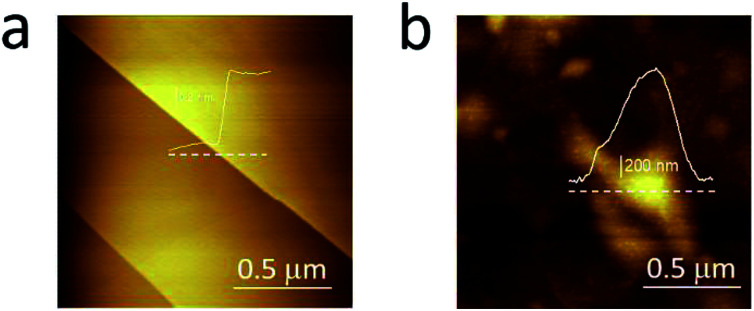
AFM images acquired in air on the pristine HOPG specimen (a) and after several EC treatments (b) to enhance the blistering process.

In Fig. 3, we compare the morphology (in air) of the pristine graphite specimen (panel a) and that one collected after several CVs (panel b). In the latter, blisters affect the surface and the overall roughness is increased, due to the carbon dissolution caused by high electrochemical applied potentials.

**Fig. 4:**
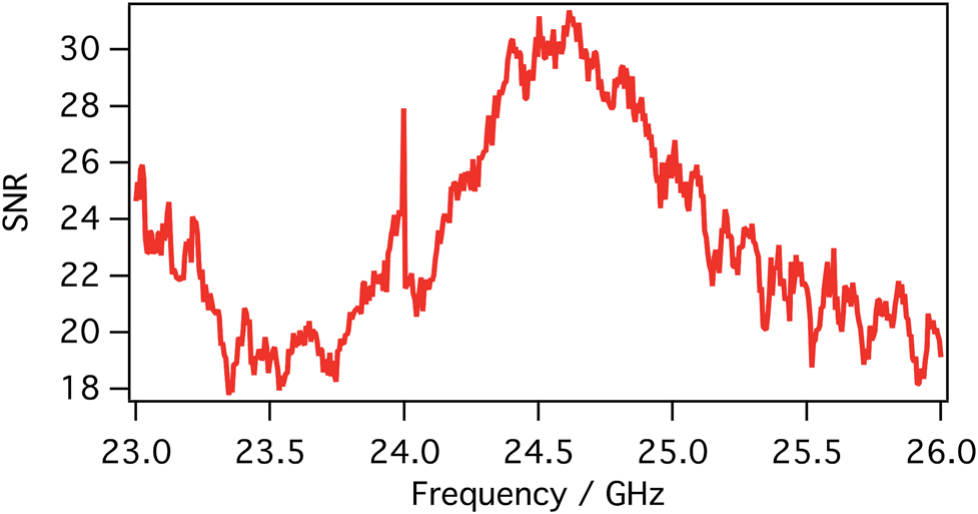
Signal-to-noise (in dB) ratio obtained between 23 and 26 GHz.

SMM data are recorded in a frequency band where the signal-to-noise ratio (SNR) is higher than compared to other frequencies. SNR is defined in the following eqn (1):1
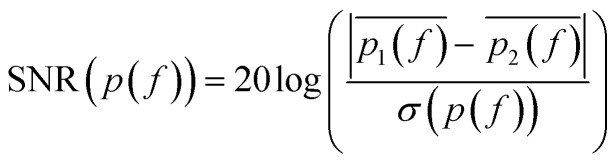
where *p*(*f*) are the complex number microwave reflection coefficients *S*_11_; *p*_1_(*f*) and *p*_2_(*f*) are measured under two distinct conditions: (i) when the tip and the sample are in close proximity (the tip is approached) and (ii) when the tip and the sample are separated by 1–2 μm; this latter situation is obtained by switching off the feedback with a corresponding change in probe–sample distance. In order to reduce the noise, the measurement was repeated for each condition 500 times to get the average *p*_1_(*f*) and *p*_2_(*f*), as well as the standard deviation *σ*. The definition of SNR accounts for both the amplitude and phase of the scattering parameters due to their appearance in a complex form in eqn (1), where the distance in the complex plane is evaluated. Usually acceptable frequency-domain images were obtained with SNR > 20 dB. The SNR between 23–26 GHz obtained in our case is reported in Fig. 4; this frequency range has been used as working band thanks to the high response of the system.

**Fig. 5:**
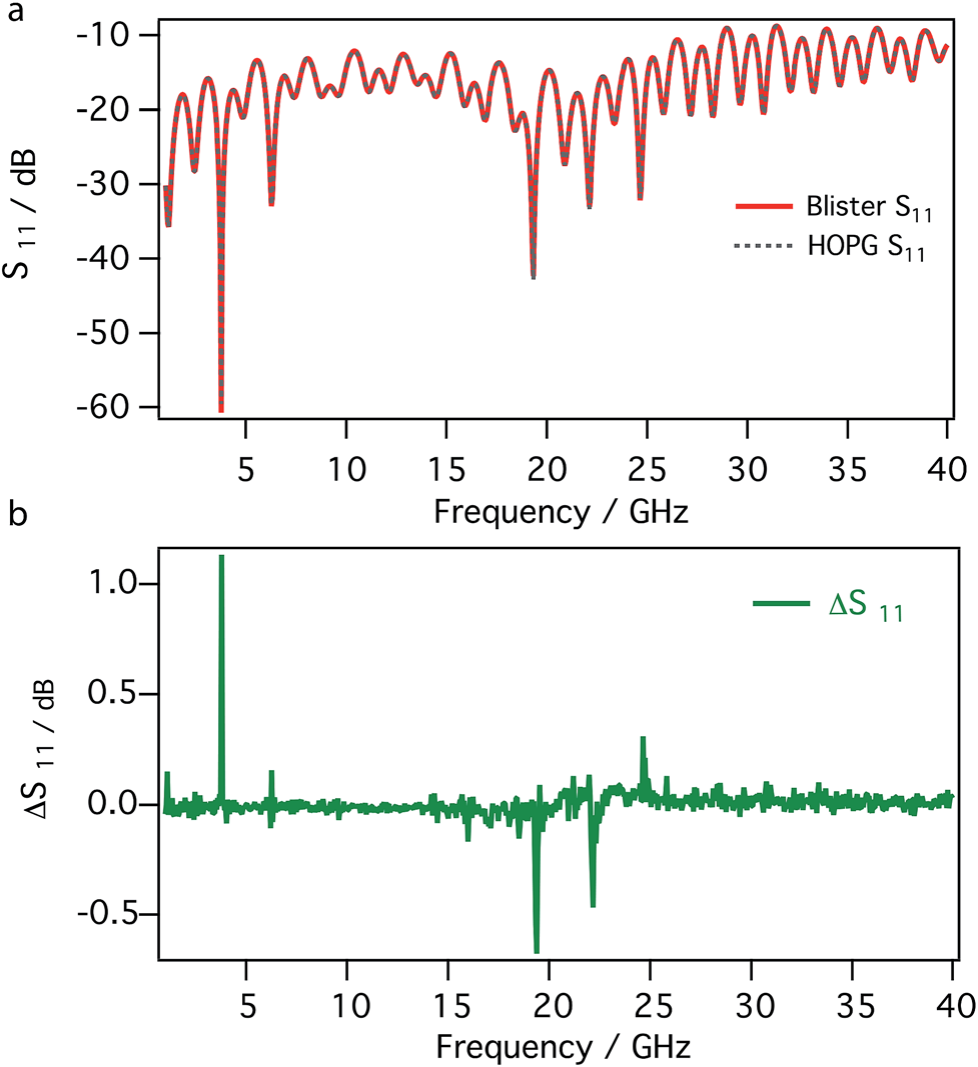
Reflection coefficients (magnitude of *S*_11_) between 1 and 40 GHz recorded on a blister and on bare HOPG (a) and their difference (b).

Fig. 5 compares the detailed frequency dependence, between 1 and 40 GHz, of the reflection coefficients (magnitude of *S*_11_ in dB) measured on the surface of blister and on bare HOPG. The *S*_11_ signals recorded in different region of the sample, *e.g.* on the blister and on the untreated HOPG, are almost completely overlapped; as shown by the difference between the signals (Fig. 5b), only minor changes are present. This is not surprising, given the nature of the material, *i.e.* the surface of the blister is made by few layers of graphene as the untreated HOPG.

**Fig. 6:**
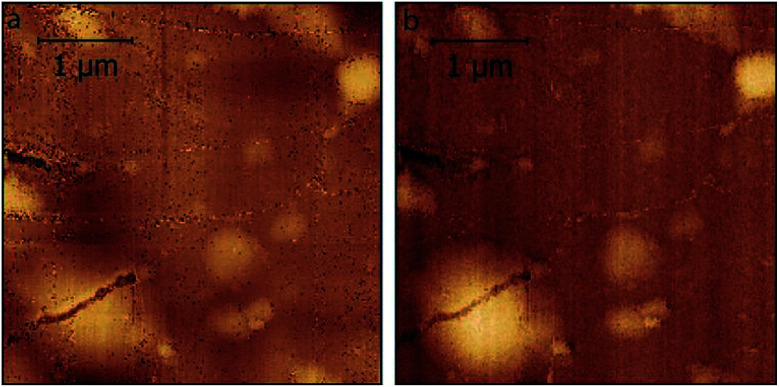
STM image (a) and simultaneous SMM image in time-domain (b) (dimension 4 μm × 4 μm in *x* and *y*).

**Fig. 7:**
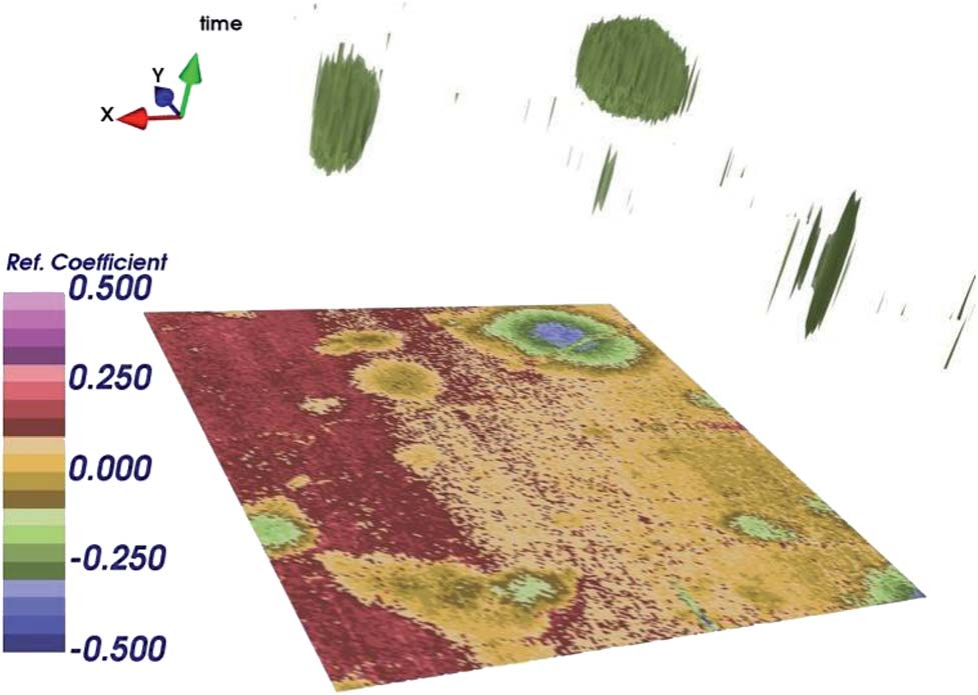
3D image obtained by plotting surface in time domain having the same reflection coefficient *S*_11_ (isosurfaces), after renormalization, in arbitrary units.

As already mentioned, simultaneous STM/SMM images were acquired on treated HOPG through a 256 × 256 scan at 512 frequencies from 23 to 26 GHz. Fig. 6 shows the STM image (a) and the SMM image after the time domain post-process (b). Compared to the STM, the SMM image have comparable spatial resolution but reveals more details of the surface, demonstrating that the SMM brings to an improvement of the image quality. Moreover, the STM image was corrected for the sample tilt (plane removal) during the post processing, whereas the SMM images did not need any graphical correction. This is of fundamental importance in order to keep the details of the surface unaltered.The SMM image of Fig. 6b can be replotted at different reflection times, as shown in Fig. 7. Thanks to the ability of the SMM technique to penetrate to some extent under the sample surface, the 3D image is obtained in post-processing from the data cube (*x*, *y*, time), by plotting surfaces having the same reflection coefficient (isosurfaces). To obtain visually effective images, average is removed from all time frames and the image is scaled, so that the reflection coefficient displayed is in arbitrary units. Even if it is not possible to give information about the number of layers on the top of the blister (the 3D image cannot be seen as a true depth profile because the data are convolved with the external structure), the presence of a 3D blister is confirmed in the region where both the STM and the SMM detected a convex folding of the HOPG. This confirms the ability of the SMM for the sub-surface characterization and helps to visualize the blisters formed as a consequence of the electrochemical process.^11^

## Conclusions

In this work we presented a characterization of the HOPG that underwent electrochemical process in acid electrolyte solution. As describe in the details, the CV involves the formation of blister under the surface of graphite. To ensure a substantial damage of the pristine graphite, the CV was repeated few times and the surface was imaged by AFM.

By using our home-made STM assisted SMM, we compared the reflection coefficients *S*_11_, between 1 and 40 GHz, recorded on the surface of the blister and on the bare HOPG and we recorded the simultaneous STM/SMM images. After the time domain post-process, the SMM image has a comparable spatial resolution but reveals more details of the surface than the STM one. The improvement that can be obtained with such microscopy technique regards not only the imaging, but also the needless of graphical post-processing; moreover, the SMM allows a sub-surface characterization as demonstrated by the 3D image, obtained by plotting surfaces having the same reflection coefficient (isosurfaces).

The SMM characterization of the graphite is supportive for a better understating of the above described phenomena and it brings to a three-dimensional view of the sub-layer structure.

## Conflicts of interest

There are no conflicts to declare.

## Supplementary Material
